# Case Report: Shark bite resulting in a urethral obstruction and urinary tract-body wall fistula in a bottlenose dolphin (*Tursiops truncatus*) in Sarasota Bay, Florida

**DOI:** 10.3389/fvets.2025.1551129

**Published:** 2025-05-09

**Authors:** Krystan A. Wilkinson, Jennifer N. Langan, Jennifer M. Meegan, Christina N. Toms, Robyn Faulkner Allen, Jay C. Sweeney, Deborah A. Fauquier, Jeremy J. Kiszka, Eric T. Hostnik, Ashley Barratclough, Michael T. Walsh, Randall S. Wells

**Affiliations:** ^1^Sarasota Dolphin Research Program, Brookfield Zoo Chicago ℅ Mote Marine Laboratory, Sarasota, FL, United States; ^2^Brookfield Zoo Chicago, Animal Health, Welfare and Science, Brookfield, IL, United States; ^3^Department of Veterinary Clinical Medicine, College of Veterinary Medicine, University of Illinois, Urbana, IL, United States; ^4^National Marine Mammal Foundation, San Diego, CA, United States; ^5^New College of Florida, Sarasota, FL, United States; ^6^Dolphin Quest, San Diego, CA, United States; ^7^National Oceanic and Atmospheric Administration, National Marine Fisheries Service, Office of Protected Resources, Silver Spring, MD, United States; ^8^Department of Biological Sciences, Institute of Environment, Florida International University, North Miami, FL, United States; ^9^Department of Clinical Sciences, Ohio State University College of Veterinary Medicine, Columbus, OH, United States; ^10^Department of Comparative, Diagnostic and Population Medicine, College of Veterinary Medicine, University of Florida, Gainesville, FL, United States

**Keywords:** ultrasound, health assessment, cetacean, marine mammal, trauma, radiograph, elasmobranch, tiger shark

## Abstract

A juvenile male common bottlenose dolphin (*Tursiops truncatus*) was examined as part of a long-term dolphin research and monitoring program in Sarasota Bay, Florida. Scars consistent with a shark bite, identified as a possible tiger shark (*Galeocerdo cuvier*), were observed at the proximal ventral peduncle bilaterally, involving the area at the distal genital slit. A left ventrolateral urinary tract-body wall fistula was identified at the cranial margin of the healed shark bite scar. The area was closely associated with palpable scar tissue at the base of the penis within the genital slit. Physical and ultrasonographic examination and attempts at urinary catheter placement supported findings of a urethral stricture with a urethral or vesicocutaneous fistula. Hematuria was detected on urinalysis, and mild hydronephrosis and lymphadenopathy were observed via ultrasonography. Despite experiencing substantial soft tissue trauma from the shark bite and subsequently developing a urinary tract obstruction with fistula formation, this animal has maintained good body condition since the health exam. Due to the location of the urethral obstruction and fistula, this animal may not be able to reproduce if it survives to breeding age. Long-term prognosis will likely be determined by the sequelae of potential progressive hydronephrosis. This case report documents a rare medical condition as a result of a shark bite not previously described in a free-ranging bottlenose dolphin, including unique historical and ensuing behavioral/health data, which is rarely possible when monitoring free-ranging wildlife.

## Introduction

Common bottlenose dolphins (*Tursiops truncatus*) are widely distributed in estuarine and coastal marine ecosystems and are valuable sentinels to assess marine ecosystem health (1). Some populations have been closely monitored over multiple decades, the results of which have greatly expanded our knowledge of dolphin health, biology, and population structure and have identified environmental and anthropogenic stressors affecting their survival. The long-term resident dolphins of Sarasota Bay, Florida, United States, have been studied by the Sarasota Dolphin Research Program (SDRP) since 1970 (2). The individually identifiable residents are monitored through monthly systematic photo-identification surveys throughout the community range that extends from southern Tampa Bay to Venice, Florida (2). The community consists of approximately 170 individuals, studied over at least six generations, spanning as many as five concurrent generations within a given maternal lineage, and includes individuals up to 67 years of age (3). Periodic catch-and-release health assessments (1) supplement photo-identification survey data with background life history, morphometric, and health information (4). During a 2023 health assessment, a juvenile male bottlenose dolphin was examined and found to have a large, healed shark bite over the proximal ventral peduncle and genital slit associated with a urinary tract-body wall fistula.

Urinary tract fistulas can be congenital or acquired due to underlying pelvic disease or trauma. Lower urinary tract fistulas can occur as uroenteric, urogenital, and urocutaneous fistulas, including vesicocutaneous (VCF, bladder to skin) (5) and urethrocutaneous (urethra to skin) forms. These fistulas develop when the ureter, bladder, or urethra forms a connection or tract with an adjacent structure, allowing urine leakage from the urinary system. Congenital forms (urogenital > urorectal fistulas) (6–9) have been uncommonly reported in dogs (10–12), cats (13), cows (14), sheep (15), and horses (16). Acquired forms generally involve any inflammatory process that compromises the lower urinary tract. The most common causes include urinary calculi, infection, neoplasia, trauma, iatrogenic, and radiation therapy (5). Clinical signs are characterized by the type and location of the fistula, typically including urine leakage through single or multiple discharge sites outside the body through the skin, reproductive, or lower gastrointestinal tract. Genitourinary symptoms may include local or abdominal pain, changes in urinary frequency, dermatitis at the leakage site, recurrent cystitis, and can lead to life-threatening infections, including sepsis. Visualization of the exact location and path of a urinary system fistula often requires advanced imaging for confirmation. While nephrolithiasis (17, 18), ureteral obstruction (19), prostatitis (20), patent urachus (21), and cystitis (22, 23) have been reported in dolphins, to the authors’ knowledge, no previous reports of urinary fistulas have been reported in this taxon.

## Case description

**Figure 1:**
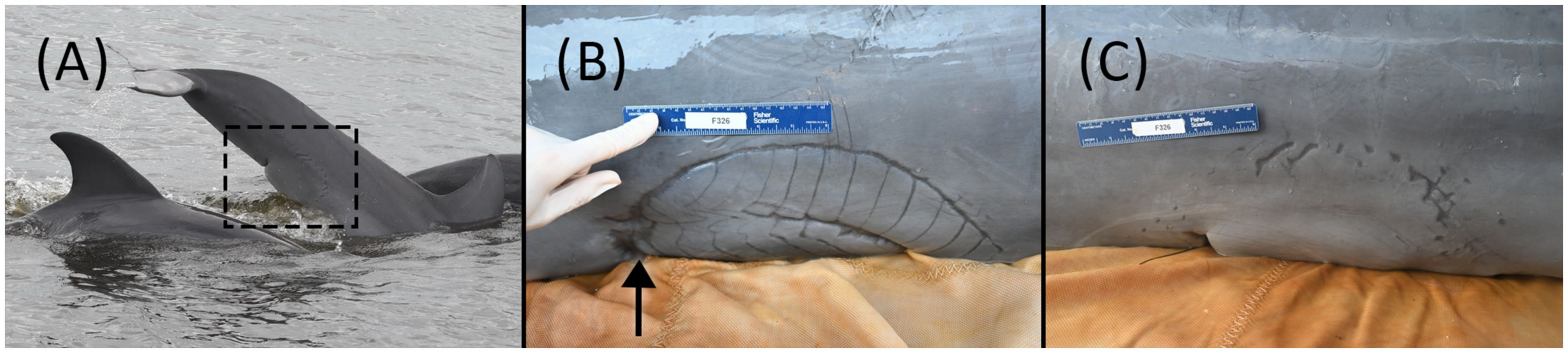
**(A)** First boat-based observation of F326 “Squirt” on 14 November 2022. The shark bite was present and healed at the time of the first sighting. **(B)** Shark bite wound over the left-side proximal ventral peduncle and genital slit during the health assessment examination on 12 May 2023. Urine was intermittently projected from a 1 mm pinpoint circular defect in the skin (arrow shows approximate location), consistent with a fistula, at the cranial portion of the shark bite scar. **(C)** Shark bite wound on the right-side proximal ventral peduncle and genital slit during the health assessment examination on 12 May 2023.

**Figure 2:**
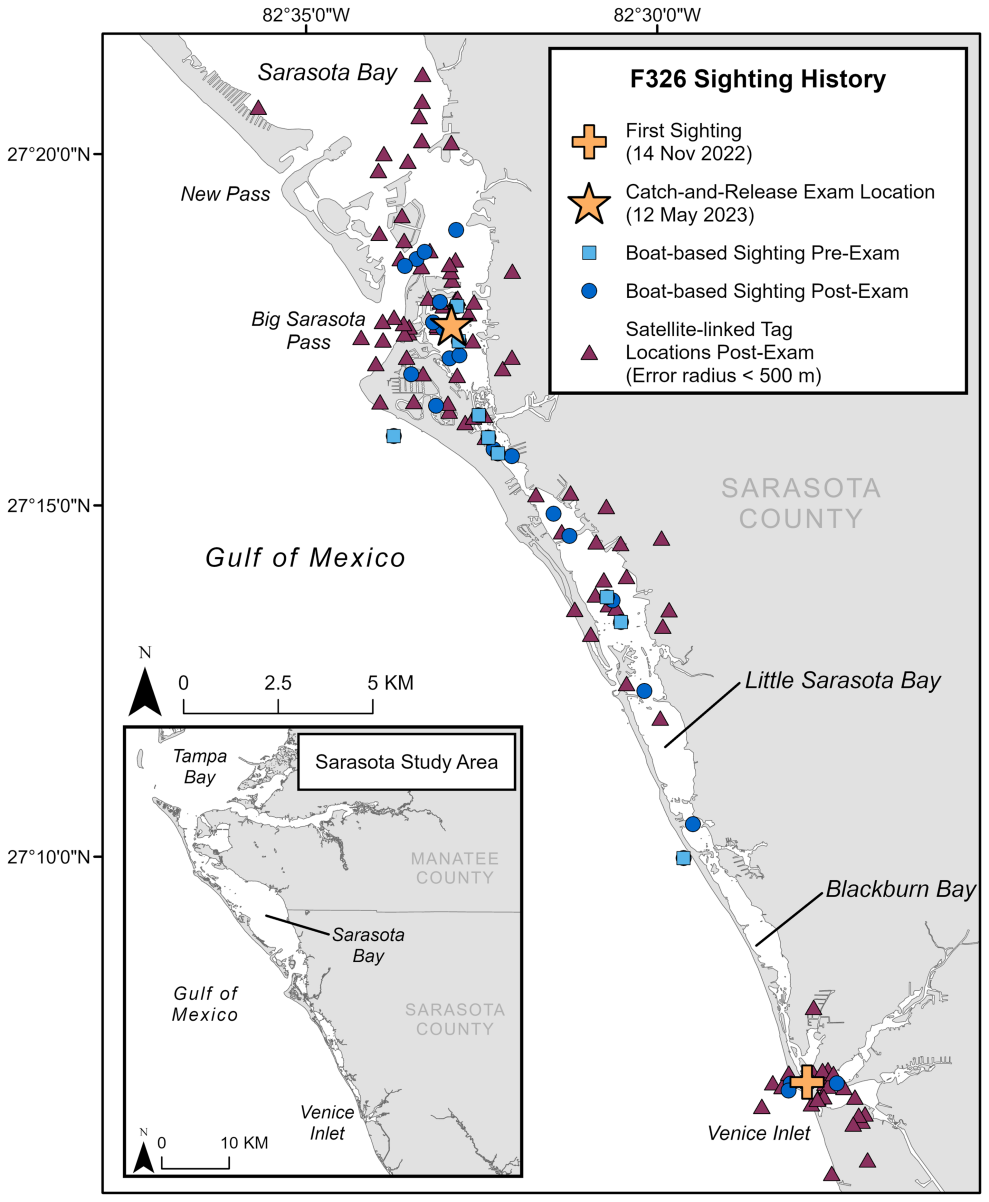
Sighting history for F326 “Squirt,” including first sighting location in the study area, location of catch-and-release health exam, locations during monthly photo-identification boat-based surveys pre- and post-examination (through November 2024), and satellite-linked tag locations post-examination.

A 126.6 kg, approximately 4.4-year-old (radiographically determined age (24); see Supplementary material 1), male bottlenose dolphin (222 cm long from tip of the upper jaw to the fluke notch; maximum girth of 123 cm), from Sarasota Bay, Florida (27.29282, −82.54878), was evaluated during population health assessments on 12 May 2023. Photographic identification of the animal’s dorsal fin through systematic monthly surveys identified that this animal entered Sarasota Bay approximately 6 months prior to the health assessment (first sighting on 14 November 2022). It is unknown where the dolphin originated, as resident communities of bottlenose dolphins are found along the western coast of Florida—such as Charlotte Harbor to the south (25) and Tampa Bay to the north of Sarasota (26)—and in near-and-offshore waters in the Gulf of Mexico (48). Photo documentation from the initial sighting confirmed the presence of a large, healed scar consistent with a shark bite on the peduncle (Figure 1A). Prior to the examination, the animal had been observed across 10 survey days from 14 November 2022 to 09 May 2023, primarily in the southern portion of the Sarasota study area (Figure 2). Observations from survey vessels documented the animal in groups of 1–13 dolphins (average = 5) and engaging in normal feeding and social behavior.

The methodology described in Wells et al. (1) was used to perform a hands-on physical examination and diagnostic sampling (NOAA/NMFS Scientific Research Permit #26622). The animal was calm and amenable to handling initially in the water and subsequently on a soft, padded, and shaded mat on the veterinary examination vessel. On physical examination, the dolphin was bright, alert, and responsive, with no abnormal findings in heart rate, respiratory rate, or mucous membranes. The most significant finding was a semicircular healed scar (35 × 11 cm) on the ventral and lateral proximal peduncle, consistent with a shark bite (Figures 1B,C). Individual longitudinal scars (1.5–2.5 cm apart) were demarcated on the left side of the peduncle, with two clear semicircular scars, one inside the other. On the right side, the scar was less defined, having one semicircular scar of similar size. The cranial margin of the bite wound scar was the widest on the left cranial aspect and coursed across the distal genital slit (base of the penis) bilaterally. The caudal margin of the smaller semicircle on the left was associated with a 2 × 2 cm defect in the ventral peduncle. At the cranial portion of the semicircular scar on the left ventral body wall, there was a 1 mm pinpoint circular defect in the skin and underlying soft tissue from which yellow fluid (urine) projected intermittently in a stream from the body, consistent with a urethral or vesicocutaneous fistula based on its location (see Supplementary video 1). Off-white caseous debris was present within the genital slit, covering the distal 4 cm of the tip of the penis. On palpation within the caudal genital slit, firm scar tissue was present at the base of the penis. Attempts to pass a red rubber catheter (5 Fr Kendall, Covidien, Mansfield, MA and 8 Fr Kendall, Cardinal Health, Waukegan, IL) for routine sterile urine collection were unsuccessful, as the catheter could only be advanced 12 cm into the distal urethra, suggesting a distal urethral stricture or obstruction. Urine flow was not observed from the genital slit, penis/urethra, at any time during the examination.

**Figure 3:**
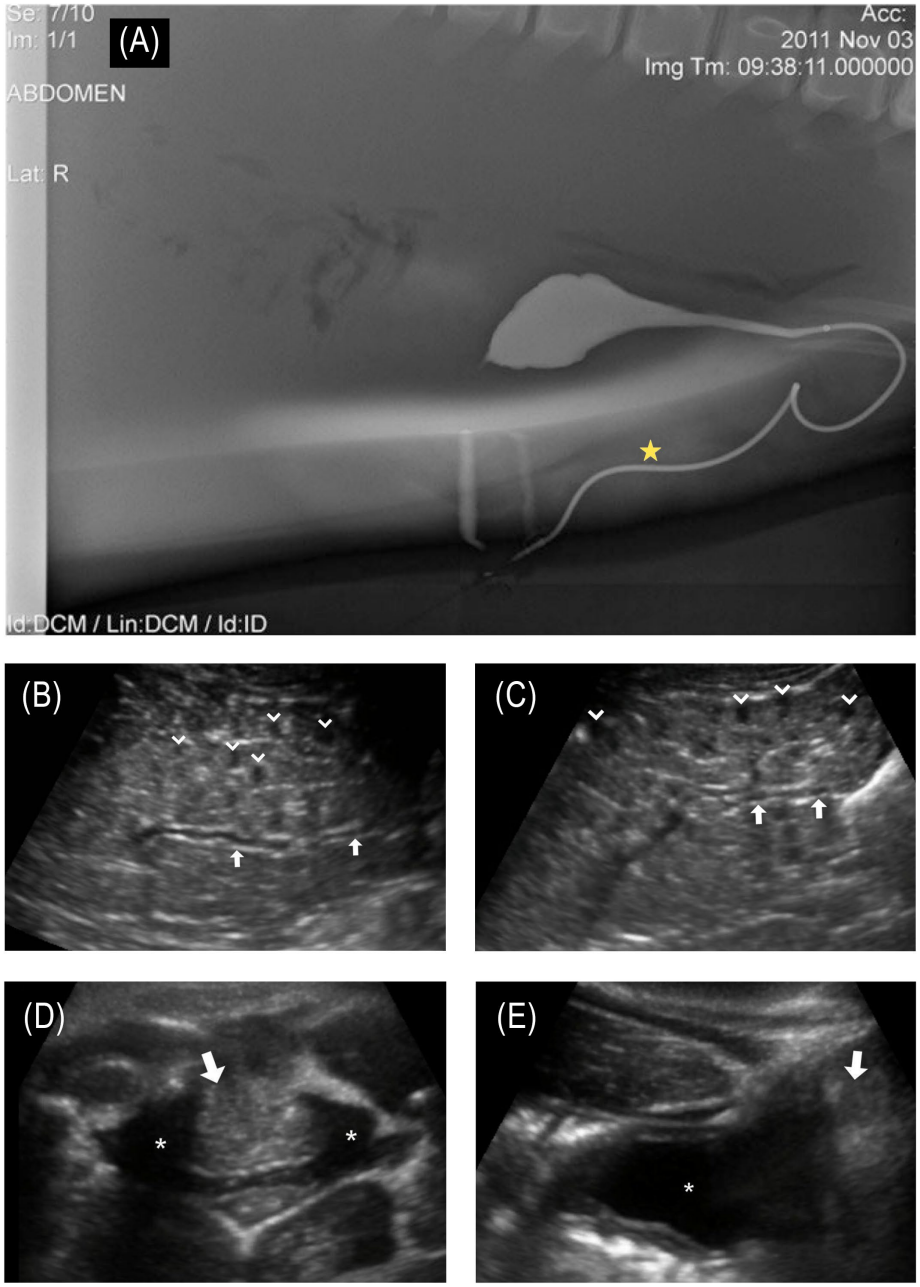
**(A)** © Sur et al. (17); Published by Mary Ann Liebert, Inc. Normal male bottlenose dolphin retrograde cystourethrogram demonstrates normal bladder and urethral anatomy; of note, the urethra has a marked curvature at the base of the sigmoid flexure. The star denotes the approximate location of the fistula that was observed in the affected animal. **(B)** Longitudinal ultrasound of the left kidney and **(C)** right kidney. Collecting ducts are slightly distended (arrows in images **B,C**). Multifocal reniculi with mild hydronephrosis are visible (chevrons); **(D)** transverse and **(E)** longitudinal ultrasound images centered on the urinary bladder (asterisk in images **D,E**). Along the caudodorsal wall of the bladder, there was a hyperechoic structure (arrows in images **D,E**), bulging into the trigone of the bladder wall, creating a mass effect. This mass-like structure (2.8 cm wide × 5.8 cm length) appeared to extend dorsolaterally through the mid-penile shaft and extended to the blubber skin layer of the lateral body wall.

A full-body in-water ultrasound was performed in sternal recumbency. Ultrasound of the urinary tract revealed bilaterally symmetrical kidneys considered to be normal in size for this species and aged animal (maximum diameter: L = 6.7 cm; R = 6.5 cm). There were focal areas of mild hydronephrosis within individual renicules that were distended with anechoic fluid (< 30 renicules affected per kidney; Figures 3B,C). The collecting ducts were mildly distended bilaterally (maximum diameter: L = 3.1 mm; R = 3.6 mm). No nephroliths were identified. Along the caudodorsal wall of the bladder, there was a hyperechoic structure bulging into the dorsal trigone of the bladder wall, creating a mass effect (Figures 3D,E). This mass-like structure (2.8 cm wide × 5.8 cm long) appeared to extend dorsolaterally through the mid-penile shaft and extended to the blubber skin layer of the lateral body wall. The forestomach was moderately distended with a mixture of whole fish and fluid of mixed echogenicity, indicating that the animal had recently foraged. The remainder of the organs examined were unremarkable.

Dorsoventral and lateral radiographs of the thorax, vertebrae, pectoral flipper, and peduncle/pelvic region were acquired on board the examination vessel. Radiographs of the thorax and skeleton were unremarkable. Irregular undulation margins of the ventral cutaneous body wall margin consistent with the bite wound/scar tissue were observed.

Point of care venous blood gas showed the animal to have mild respiratory and metabolic acidosis with appropriate systemic compensation and increased glucose (Supplementary Table 1) over the course of the examination, consistent with a stress response. Complete blood count, serum biochemical analysis, protein electrophoresis (EPH), iron panel, thyroid hormones, and cortisol were within reported reference ranges with no indication of renal compromise (Supplementary Table 1) (27–30). Testosterone was consistent with prepuberty (Supplementary Table 1) (31). The urinalysis was unremarkable except for having a moderate amount of blood on the dipstick and a large amount of amorphous debris on cytological examination (32). Urine cultures were not submitted. The cytology of blowhole, gastric, and fecal samples was unremarkable.

Prior to release, the dolphin received a freeze brand (326) for long-term identification and a Wildlife Computers SPLASH10 satellite-linked finmount tag for tracking his movements. The observation of urine intermittently squirting from the side of the body led to this dolphin being referred to as “Squirt.”

## Discussion

We considered the injury to be shark-inflicted based on the crescent shape of the wound and the presence of widely spaced teeth impressions. The identification of predator species involved in non-lethal lesions on dolphins is challenging. However, the size (particularly the width) and shape of the wound can be used to identify the predator species involved (33–35). Bull sharks (*Carcharhinus leucas*) and tiger sharks (*Galeocerdo cuvier*) are common predators of the Sarasota dolphin community (36–38) and in the nearshore Gulf of Mexico coastal areas; though as previously mentioned, this bite occurred prior to the animal moving into the Sarasota study area, and it is unknown from where the animal originated. Dusky sharks (*Carcharhinus obscurus*) are also found in the Gulf of Mexico, as are occasional white sharks (*Carcharodon carcharias*), both of which are also considered dolphin predators (33, 36–39). Carcharhinid shark bites (e.g., bull and dusky sharks) are crescent-shaped and clean-cut, while white sharks are wider and parabolic (33, 34). Tiger shark bites are broad in shape and tend to leave widely spaced rake marks (34), consistent with what was observed on this animal. The longitudinal scars were measured 1.5–2.5 cm apart, larger than the interdental distance of bull or dusky sharks but within the range of tiger sharks (35). Though scar growth over time cannot be ruled out, the wound’s shape, widely spaced rake marks, and three independent expert opinions indicate the predator was most likely a tiger shark.

Free-ranging cetaceans can survive tremendous trauma and physical challenges, both natural (e.g., shark bites, harmful algal blooms, hurricanes) and anthropogenic (e.g., entanglement, boat strikes, and oil spills); however, these threats can also result in substantial morbidity and mortality. Long-term monitoring, research, and health assessments of wild dolphin populations, such as the community in Sarasota Bay, have provided critical global reference data essential for evaluating at-risk populations and investigating causes of mortality [e.g., (40–43)]. While most common bottlenose dolphin populations are not currently considered threatened, and the vast majority of dolphins in Sarasota Bay are in good health, the loss of even a single animal may have significant effects on a population at the local level (44). The animal described in this case study survived the initial shark interaction; however, the resulting wounds and subsequent scarring have most likely prevented any possibility of this animal successfully reproducing if/when it reaches sexual maturity. Monthly photographic surveys have proven to be a valuable tool for documenting population dynamics, serving to identify new individuals or the disappearance of well-known ones within this population. These efforts identified this animal as a new addition to the local community and documented its survival from a sizable shark bite. Following the health assessment, the animal was observed on more than 20 boat-based photographic surveys, in group sizes ranging from 1 to 11 dolphins (average 4.38) and engaging in typical feeding and social behavior (at the time of this communication, November 2024, Figure 2). Although the satellite-linked tag was shed after approximately 3.5 months (as designed), as of this writing, the dolphin spends a great deal of time in inshore waters and canals where it is reported by the public, often with photo documentation from shore. Post-health assessment sightings, public reports, and satellite-linked locations confirm that the animal has stayed in the southern part of the Sarasota study area, which is consistent with his sighting history prior to catch-and-release sampling (Figure 2). The dolphin is readily identifiable by its freeze-brand and will continue to be monitored during monthly SDRP photo-identification surveys.

Thorough hands-on health evaluations of individuals in a population can detect findings that are not apparent from observations alone (such as disease prevalence, immune dysfunction, contaminant levels, and individual health), as demonstrated by the identification of a urinary fistula in this animal. While it is not possible to determine exactly when the injury occurred, it is likely that it happened several months prior to the initial sighting, based on the wound being fully healed. Had this animal not developed a fistula to allow urine passage, it likely would have succumbed to the sequelae of urinary obstruction (17–19, 45) despite recovering from the wounds associated with a shark bite. Examination findings of a urethral stricture/obstruction, along with a small-diameter urethral or vesicocutaneous fistula, would prevent the animal from fully emptying its bladder of urine, likely contributing to the mild hydronephrosis and dilated collecting ducts identified on ultrasonographic examination. Hematuria may be due to chronic trauma to the bladder, urethra, or leakage from the tissue making up the fistulous tract. In comparing the location of the fistula to cross-sectional anatomy from contrast-enhanced computed tomography (CT) and previously published cystourethrogram (Figure 3A), the location of the fistula is most likely associated with a urethral fistula but a vesicocutaneous tract cannot be ruled out.

While not possible in this case, imaging contrast studies, such as CT and magnetic resonance imaging (MRI), are the most sensitive diagnostic techniques for identifying fistulas and associated abnormalities. Additionally, a fistulogram, urethroscopy, cystoscopy, and ureteroscopy can also be helpful in diagnosing a fistula in some cases. Despite the unavailability of these advanced diagnostic modalities in this case, physical examination, diagnostic testing, and ultrasonographic imaging allowed for the diagnosis of this animal’s urethral/vesicocutaneous fistula. Abdominal and pelvic ultrasound is one of the most widely used modalities for diagnosing urinary tract disease, but it can be more difficult to identify fistulous tracks with this modality. In this case, the ultrasound detected abnormalities closely associated with the bladder and base of the penis (Figures 3D,E) at the level of the scars from the shark bite; however, the fistula itself would not have been identified without an out-of-water examination, demonstrating the value of a thorough physical evaluation as part of the complete health assessment process. The location of the fistula, the yellow color of the fluid, the expected urinalysis results, and the shrinking size of the bladder after fluid excretion from the fistula confirmed the fluid to be urine and the findings to be consistent with the animal having a urethral or vesicocutaneous fistula.

Hematological and serum biochemical parameters, as well as most urinalysis findings, were within reference intervals for bottlenose dolphins from this geographical site, supporting that the animal did not have a systemic infection or localized cystitis at the time of the examination (29). These findings support that there is unlikely to have been any ongoing ascending leakage of saltwater retrograde up the fistula. Total protein, albumin, sodium, PCV, and BUN values (Supplementary Table 1) were at the upper threshold of the range for juveniles (29), suggesting that this animal was mildly dehydrated. Additionally, there was evidence of recent consumption of a high-protein meal, which may have also contributed to these findings. Alkaline phosphatase was also within the upper threshold of values (29, 46), supporting a lack of acute illness (46, 47) and may be associated with growth.

Treatment was not possible for this individual or generally for free-ranging dolphins. Treatment and rehabilitation for ill, injured, or stranded dolphins are rare and determined through collaborative local, regional, and federal response agencies, their capabilities, and species management plans. For people, domestic species, and dolphins in managed care, CT, including a contrast urethrogram, urethroscopy, reestablishing urethral patency, and repair of the fistula, would be recommended or considered.

The prognosis for this dolphin with urethral obstruction, urethral/vesicocutaneous fistula, and early signs of post-renal urinary obstruction (mild hydronephrosis, dilated collecting ducts) is unknown. The animal survived initial recovery from a substantial shark bite from which trauma and scar tissue resulted in the findings described in this report. While this juvenile has maintained good body condition and did not have findings consistent with ascending infection or azotemia (elevated renal values), it is likely that continued chronic increased pressure in the bladder, due to the inability to urinate normally, will result in continued increased retrograde pressure through the ureters to the kidneys that will likely cause progressive renal compromise over time. As long as the animal has a fistula, the risk of ascending infection will remain increased. Additionally, reproductive fluids, including semen, will also not be able to pass, prohibiting this animal from successful reproduction.

This case highlights how attempted predation events can have enduring impacts on a local dolphin community, even when the dolphin survives the predation attempt. It also illustrates the sometimes-exceptional abilities of small cetaceans to recover from major traumatic injuries while underscoring the importance of long-term monitoring in conservation studies.

## Data Availability

The original contributions presented in the study are included in the article/Supplementary material, further inquiries can be directed to the corresponding authors.

## References

[1] Wells RS Rhinehart HL Hansen LJ Sweeney JC Townsend FI Stone R. Bottlenose dolphins as marine ecosystem sentinels: developing a health monitoring system. EcoHealth. (2004) 1:246–54. doi: 10.1007/s10393-004-0094-6, PMID: 40175859

[2] Wells RS. Learning from nature: bottlenose dolphin care and husbandry. Zoo Biol. (2009) 28:635–51. doi: 10.1002/zoo.20252, PMID: 19434729

[3] Wells RS. The Sarasota dolphin research program in 2020: celebrating 50 years of research, conservation, and education. Aquat Mamm. (2020) 46:502–3. doi: 10.1578/AM.46.5.2020.502

[4] Wells RS. Social structure and life history of bottlenose dolphins near Sarasota Bay, Florida: insights from four decades and five generations In: Yamagiwa J Karczmarski L, editors. *Primates and cetaceans*. Primatology monographs. Tokyo, Japan: Springer (2014). 149–72.

[5] Vázquez Gálvez A Blas Reina A García Martínez FE Figueroa Sánchez TO Arriaga J. Vesicocutaneous Fistula In: Sotelo R Polotti CF Arriaga J, editors. Urinary fistula. Cham, Switzerland: Springer International Publishing (2022). 71–81.

[6] Suess RP Martin RA Moon ML Dallman MJ. Rectovaginal fistula with atresia ani in three kittens. Cornell Vet. (1992) 82:141–53. PMID: 1623727

[7] Rahal SC Vicente CS Mortari AC Mamprim MJ Caporalli EHG. Rectovaginal fistula with anal atresia in 5 dogs. Can Vet J. (2007) 48:827–30. doi: 10.17221/8769-VETMED PMID: 17824325 PMC1914316

[8] Embertson RM. Selected urogenital surgery concerns and complications. Vet Clin North Am Equine Pract. (2008) 24:643–61. doi: 10.1016/j.cveq.2008.10.007, PMID: 19203706

[9] Gangwar AK. Congenital anomalies and their surgical correction in ruminants. Adv Anim Vet Sci. (2014) 2:369–76. doi: 10.14737/journal.aavs/2014/2.7.369.376

[10] Goulden B Bergman MM Wyburn RS. Canine urethrorectal fistulae. J Small Anim Pract. (1973) 14:143–50. doi: 10.1111/j.1748-5827.1973.tb06909.x, PMID: 4732949

[11] Chandler JC MacPhail CM. Congenital urethrorectal fistulas. Compend Contin Educ Pract Vet. (2001) 23:995–1002.

[12] Thompson JL Liuti T Albuquerque C Murgia D. Congenital Urethrovaginal fistula with blind-ending vagina in a female Pseudohermaphrodite dog with urinary incontinence. J Am Anim Hosp Assoc. (2021) 57:237–41. doi: 10.5326/JAAHA-MS-7114, PMID: 34370856

[13] John MK Cari AO Jody PL Rodney EO. Inherited and congenital diseases of the feline lower urinary tract. Vet Clin Small Anim Pract. (1996) 26:265–79. doi: 10.1016/S0195-5616(96)50207-X, PMID: 8711862

[14] Özak A Hö N Yardimci C Kabak YB. Anorectal malformation with Colovesical and Colourethral fistula in two calves. Kafkas Univ Vet Fak Derg. (2015) 21:287–9. doi: 10.9775/kvfd.2014.12166

[15] Vahar M Hosseni SM Omidzahir S Kenari EO Iraee MA Ziabari AH. Report of congenital colonobladder fistula with atresia ani in a lamb and treatment by surgery. Asian Pac J Trop Dis. (2015) 5:S181–3. doi: 10.1016/S2222-1808(15)60885-4

[16] Cruz AM Barber SM Kaestner SBR Townsend HGG. Urethrorectal fistula in a horse. Can Vet J. (1999) 40:122–4. PMID: 10065321 PMC1539560

[17] Sur RL Meegan JM Smith CR Schmitt T L’Esperance J Hendrikson D. Surgical Management of Nephrolithiasis in the bottlenose dolphin: collaborations between the urologist and veterinarian. J Endourol Case Rep. (2018) 4:62–5. doi: 10.1089/cren.2017.0143, PMID: 29756043 PMC5944394

[18] Meegan JM Smith CR. Chapter 82 - dolphin nephrolithiasis In: Miller E Lamberski N Calle P, editors. Fowler’s zoo and wild animal medicine current therapy, vol. 10. New Delhi: W.B. Saunders (2023). 565–72.

[19] Schmitt TL Sur RL. Treatment of ureteral Calculus obstruction with laser lithotripsy in an Atlantic bottlenose dolphin (*Tursiops truncatus*). J Zoo Wildl Med. (2012) 43:101–9. doi: 10.1638/2011-0002.1, PMID: 22448516

[20] Suárez-Santana CM Sierra E Díaz-Delgado J Zucca D De Quirós YB Puig-Lozano R. Prostatic lesions in Odontocete cetaceans. Vet Pathol. (2018) 55:466–72. doi: 10.1177/0300985818755252, PMID: 29402205

[21] Larson KM Maretelli PR Khong LF Fernando NM. Multiple birth defects in a captive-born 10-day-old indo-Pacific bottlenose dolphin (*Tursiops aduncus*) with a likely association to a head-first presentation at parturition. (2012). Available online at: https://www.vin.com/doc/?id=6699186.

[22] Reidarson TH McBain J. The combined use of Itraconazole and Flucytosine in the treatment of chronic Candida cystitis in a bottlenose dolphin (*Tursiops truncatus*). (1995). Available online at: https://www.vin.com/doc/?id=6696240.

[23] Vargas-Castro I Crespo-Picazo JL Fayos M Jiménez-Martínez MDLÁ Torre-Fuentes L Álvarez J. New insights into the pathogenesis and transmission of *Brucella pinnipedialis*: systemic infection in two bottlenose dolphins (*Tursiops truncatus*). Microbiol Spectr. (2023) 11:e01997–23. doi: 10.1128/spectrum.01997-23, PMID: 37800951 PMC10848334

[24] Barratclough A Sanz-Requena R Marti-Bonmati L Schmitt TL Jensen E García-Párraga D. Radiographic assessment of pectoral flipper bone maturation in bottlenose dolphins (*Tursiops truncatus*), as a novel technique to accurately estimate chronological age. PLoS One. (2019) 14:e0222722. doi: 10.1371/journal.pone.0222722, PMID: 31557197 PMC6762177

[25] Bassos-Hull K Perrtree RM Shepard CC Schilling S Barleycorn AA Allen JB. Long-term site fidelity and seasonal abundance estimates of common bottlenose dolphins (*Tursiops truncatus*) along the southwest coast of Florida and responses to natural perturbations. J Cetacean Res Manage. (2013) 13:19–30. doi: 10.47536/jcrm.v13i1.551

[26] Urian KW Hofmann S Wells RS Read AJ. Fine-scale population structure of bottlenose dolphins (*Tursiops truncatus*) in Tampa Bay, Florida. Mar Mamm Sci. (2009) 25:619–38. doi: 10.1111/j.1748-7692.2009.00284.x

[27] St. Aubin DJ Ridgway SH Wells RS Rhinehart H. Dolphin thyroid and adrenal hormones: circulating levels in wild and semidomesticated *Tursiops truncatus*, and influence of sex, age and season. Mar Mamm Sci. (1996) 12:1–13. doi: 10.1111/j.1748-7692.1996.tb00301.x, PMID: 40175095

[28] St. Aubin DJ. Endocrinology In: Dierauf L Gulland FMD, editors. CRC handbook of marine mammal medicine. Boca Raton, Florida: CRC Press (2001). 165–92.

[29] Schwacke LH Hall AJ Townsend FI Wells RS Hansen LJ Hohn AA. Hematologic and serum biochemical reference intervals for free-ranging common bottlenose dolphins (*Tursiops truncatus*) and variation in the distributions of clinicopathologic values related to geographic sampling site. Am J Vet Res. (2009) 70:973–85. doi: 10.2460/ajvr.70.8.973, PMID: 19645578

[30] Hart LB Wells RS Kellar N Balmer BC Hohn AA Lamb SV. Adrenal hormones in common bottlenose dolphins (*Tursiops truncatus*): influential factors and reference intervals. PLoS One. (2015) 10:e0127432. doi: 10.1371/journal.pone.0127432, PMID: 25993341 PMC4436368

[31] Robeck TR O’Brien JK Atkinson S. Chapter 11 - reproduction In: Gulland FMD Dierauf LA Whitman KL, editors. CRC handbook of marine mammal medicine. Boca Raton: CRC Press (2018). 169–208.

[32] Deming A Stacy NI Balmer BC Sweeney J Fauquier DA Wells RS. Urinalysis in free-ranging bottlenose dolphins (*Tursiops truncatus*). from Sarasota Bay, Florida: (2012) https://www.vin.com/doc/?id=6699186.

[33] Long DJ Jones RE. White shark predation and scavenging on cetaceans in the eastern North Pacific Ocean In: Klimley AP Ainley DG, editors. Great white sharks: The biology of Carcharodon carcharias. New York, NY, USA: Academic Press (1996). 293–307.

[34] Heithaus MR. Shark attacks on bottlenose dolphins (*Tursiops aduncus*) in Shark Bay, Western Australia: attack rate, bite scar frequencies and attack seasonality. Mar Mamm Sci. (2001) 17:526–39. doi: 10.1111/j.1748-7692.2001.tb01002.x, PMID: 40175095

[35] Lowry D De Castro ALF Mara K Whitenack LB Delius B Burgess GH. Determining shark size from forensic analysis of bite damage. Mar Biol. (2009) 156:2483–92. doi: 10.1007/s00227-009-1273-3

[36] Irvine B Wells RS Gilbert PW. Conditioning an Atlantic bottle-nosed dolphin, *Tursiops truncatus*, to repel various species of sharks. J Mammal. (1973) 54:503–5.

[37] Wells RS. Bringing up baby. Nat Hist. (1991) 8:56–62.

[38] Wilkinson KA Wells RS Pine WE Borkhataria RR. Shark bite scar frequency in resident common bottlenose dolphins (*Tursiops truncatus*) in Sarasota Bay, Florida. Mar Mammal Sci. (2017) 33:678–86. doi: 10.1111/mms.12385

[39] Heithaus MR. Predator–prey and competitive interactions between sharks (order Selachii) and dolphins (suborder Odontoceti): a review. J Zool. (2001) 253:53–68. doi: 10.1017/S0952836901000061

[40] Schwacke LH Smith CR Townsend FI Wells RS Hart LB Balmer BC. Health of common bottlenose dolphins (*Tursiops truncatus*) in Barataria Bay, Louisiana, following the *Deepwater horizon* oil spill. Environ Sci Technol. (2014) 48:93–103. doi: 10.1021/es403610f, PMID: 24350796

[41] Lane SM Smith CR Mitchell J Balmer BC Barry KP McDonald T. Reproductive outcome and survival of common bottlenose dolphins sampled in Barataria Bay, Louisiana, USA, following the Deepwater horizon oil spill. Proc R Soc B Biol Sci. (2015) 282:20151944. doi: 10.1098/rspb.2015.1944, PMID: 26538595 PMC4650159

[42] Smith CR Rowles TK Hart LB Townsend F Wells RS Zolman ES. Slow recovery of Barataria Bay dolphin health following the Deepwater horizon oil spill (2013-2014), with evidence of persistent lung disease and impaired stress response. Endanger Species Res. (2017) 33:127–42. doi: 10.3354/esr00778

[43] Barratclough A Wells RS Schwacke LH Rowles TK Gomez FM Fauquier DA. Health assessments of common bottlenose dolphins (*Tursiops truncatus*): past, present, and potential conservation applications. Front Vet Sci. (2019) 6:444. doi: 10.3389/fvets.2019.00444, PMID: 31921905 PMC6923228

[44] Lacy RC Wells RS Scott MD Allen JB Barleycorn AA Urian KW. Assessing the viability of the Sarasota Bay Community of Bottlenose Dolphins. Front Mar Sci. (2021) 8:788086. doi: 10.3389/fmars.2021.788086

[45] Venn-Watson S Smith C Johnson S Daniels R Townsend F. Clinical relevance of urate nephrolithiasis in bottlenose dolphins *Tursiops truncatus*. Dis Aquat Org. (2010) 89:167–77. doi: 10.3354/dao02187, PMID: 20402234

[46] Andersen S. Physiological ranges of blood-chemical parameters in captive harbour porpoise, *Phocaena phocaena* (L). Nord Vet Med. (1968) 20:267–78.

[47] Fothergill MB Schwegman CA Garratt PA Govender A Robertson WD. Serum alkaline phosphatase- changes in relation to state of health and age of dolphins. Aquat Mamm. (1991) 17:71–5.

[48] Fazioli KL Hofmann S Wells RS. Use of Gulf of Mexico coastal waters by distinct assemblages of bottlenose dolphins (*Tursiops truncatus*). Aquat Mamm. (2006) 32:212–222. doi: 10.1578/AM.32.2.2006.212

