# Supplementary material for: Case Report: Shark bite resulting in a urethral obstruction and urinary tract-body wall fistula in a bottlenose dolphin (Tursiops truncatus) in Sarasota Bay, Florida
Source: Front Vet Sci. 2025 May 9;12:1551129. doi: 10.3389/fvets.2025.1551129 (PMC12100750; doi:10.3389/fvets.2025.1551129)
Supplement: Supplementary file 1 [file Image_1.pdf]

Supplementary Material 1

Age estimation via radiographs

A dorsoventral radiograph was performed of the left pectoral flipper to facilitate age estimation. The growth plates in the radius, ulna and metacarpals were clearly visible and predominantly showed a physeal space to be present between the bones and the epiphyseal plate indicating the young age of this animal. The physeal space on the distal radius demonstrated the very initial stages of osseous bridge formation as fusion of the metaphysis and secondary ossification center commences. An estimated age of 4.4 years was obtained by scoring the pectoral flipper across the 16 locations as determined in previously published methods (1). Ultrasound imagery showed the testicles were bilaterally symmetrical (maximum diameter: 1.95 cm; length: 9.0 cm), confirming the animal was not yet mature (2). The testicles had normal echotexture and appearance.

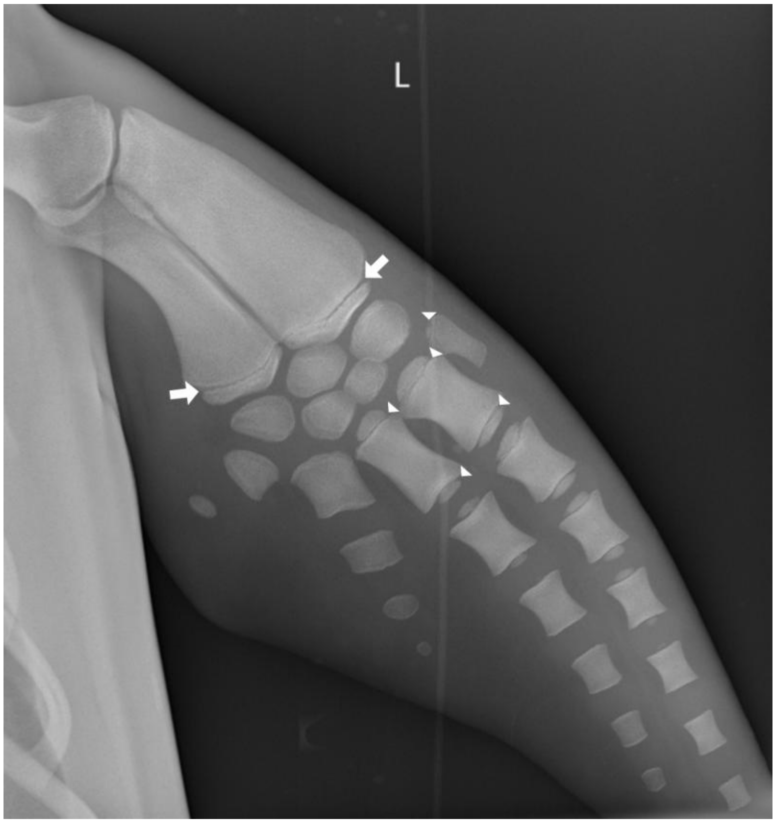

Figure 1. Dorsoventral radiograph of the left pectoral flipper. The age was estimated to be 4.4 years by scoring the pectoral flipper across the 16 locations as determined in previously published methods (1). The growth plates in the distal radius and ulna (arrows) and metacarpals (arrowheads) were clearly visible and predominantly showed a physeal space to be present between the bones and the epiphyseal plate.

Supplementary Material

References

1. Barratclough A, Sanz-Requena R, Marti-Bonmati L, Schmitt TL, Jensen E, García-Párraga D. Radiographic assessment of pectoral flipper bone maturation in bottlenose dolphins (*Tursiops truncatus*), as a novel technique to accurately estimate chronological age. *PLoS One.* (2019) 14:e0222722. doi: 10.1371/journal.pone.0222722

2. Brook FM, Kinoshita R, Brown B, Metreweli C. Ultrasonographic imaging of the testis and epididymis of the bottlenose dolphin, *Tursiops truncatus aduncas*. *J Reprod Fertil.* (2000) 119:233–240. doi: 10.1530/jrf.0.1190233
