# Supplementary material for: Case Report: Shark bite resulting in a urethral obstruction and urinary tract-body wall fistula in a bottlenose dolphin (Tursiops truncatus) in Sarasota Bay, Florida
Source: Front Vet Sci. 2025 May 9;12:1551129. doi: 10.3389/fvets.2025.1551129 (PMC12100750; doi:10.3389/fvets.2025.1551129)
Supplement: Supplementary file 2 [file Table_1.docx]

Supplementary Material 3

**Supplementary Table.** Hematology, serum biochemical, serum protein electrophoretic, urinalysis and selected point of care analyte values from a male bottlenose dolphin (Sarasota Bay, Florida) with urethral obstruction and a urinary fistula from a shark bite.

| **Parameter** | **Result** | **Reference Range** |
| --- | --- | --- |
| **Hematologic Analysis** |  | **Juvenile Reference Intervals*** |
| HCT (%) | 43 | 37-47 |
| Hemoglobin (g/dL) | 14.1 | 12.9-15.8 |
| RBC (x10^6^/cmm) | 3.6 | 3.3-4 |
| MCV (fL) | 119 | 101-127 |
| MCH (pg) | 39 | 36-44 |
| MCHC (g/dL) | 33 | 32-37 |
| RDW (%) | 11.8 | 11.3-15.9 |
| Reticulocytes (%) | 3.1 | 0.08-4.88∮ |
| Reticulocytes count (cells/µL) | 113,600 | 7,540-151,160∮ |
| WBC (cells/µL) | 7,900 | 6,400-18,100 |
| Neutrophil (%) | 46 | 31-41 |
| Bands (%) | 0 | 0 |
| Lymphocytes (%) | 29 | 17-25 |
| Monocytes (%) | 2.5 | 0-3 |
| Eosinophils (%) | 23 | 20-46 |
| Basophils (%) | 0 | 0-3 |
| Nucleated RBC | 0 | 0 |
| Neutrophils (cells/µL) | 3,600 | 2,000-7,500 |
| Bands (cells/µL) | 0 | 0 |
| Lymphocytes (cells/µL) | 2,300 | 1,100-4,500 |
| Monocytes (cells/µL) | 200 | 0-800 |
| Eosinophils (cells/µL) | 1,800 | 1,300-8,400 |
| Basophils (cells/µL) | 0 | 0-600 |
| Platelet count (platelets/µL) | 177,000 | 110,000-277,000 |
|  |  |  |
| **Serum Biochemical Analysis** |  | **Juvenile Reference Intervals*** |
| Sodium (mEq/L) | 154 | 151-158 |
| Potassium (mEq/L) | 4.1 | 3.1-4.5 |
| Chloride (mEq/L) | 118 | 107-120 |
| BUN (mg/dL) | 75 | 47-80 |
| Creatinine (mg/dL) | 0.6 | 0.7-1.5 |
| Uric acid (mg/dL) | 1.1 | 0.14-2 |
| Calicum (mg/dL) | 9.6 | 8.7-10.2 |
| Phosphorus (mg/dL) | 5.5 | 3.1-4.5 |
| Magnesium (mEq/L) | 1.5 | 1.2-1.8 |
| Total protein (g/dL) | 6.4 | 6.4-8.3 |
| Albumin (g/dL) | 4.4 | 3.8-5 |
| Globulin (g/dL) | 2 | 1.9-3.8 |
| A/G Ratio | 2.2 | 1.1-2.4 |
| Glucose (mg/dL) | 106 | 66-141 |
| ALT (U/L) | 45 | 12-70 |
| AST (U/L) | 222 | 165-371 |
| SDH (U/L) | 3 | 1-46 |
| AP (U/L) | 582 | 96-702 |
| GGT (U/L) | 23 | 12-34 |
| LDH (U/L) | 563 | 377-611 |
| Total bilirubin (mg/dL) | 0 | 0-0.2 |
| Direct bilirubin (mg/dL) | 0 | 0-0.1 |
| Indirect bilirubin (mg/dL) | 0 | 0-0.2 |
| Amylase (U/L) | <3 | 1-3^^ |
| Lipase (U/L) | 8 | 4.6-8.4∮ |
| Cholesterol (mg/dL) | 157 | 102-217 |
| Tryglycerides (mg/dL) | 113 | 47-148 |
| CK (U/L) | 251 | 104-358 |
| Fibrinogen (mg/dL) | 201 | 50-400^^ |
| Total iron (µg/dL) | 131 | 65-215 |
| TIBC (µg/dL) | 395 | 183-419 |
| Iron saturation (%) | 33 | 23-62 |
|  |  |  |
| **Protein Electrophoresis (EPH)** |  | **Juvenile Reference Intervals*** |
| Albumin (g/dL) | 3.5 | 3.1-4.3 |
| Alpha 1 globulins (g/dL) | 0.6 | 0.2-1.2 |
| Alpha 2 globulins (g/dL) | 0.4 | 0.6-1.3 |
| Total Alpha globulins (g/dL) | 1 | 1.1-1.8 |
| Beta 1 globulins (g/dL) | 0.3 | 0.11-0.35** |
| Beta 2 globulins (g/dL) | 0.2 | 0.16-0.43** |
| Total Beta globulins (g/dL) | 0.5 | 0.4-0.7 |
| Gamma globulins (g/dL) | 1.4 | 0.8-3 |
| Total globulins (g/dL) | 2.9 | 2.7-4.9 |
| A/G Ratio | 1.22 | 1.1-2.4 |
| Total protein (g/dL) | 6.4 | 6.5-8.3 |
| Cortisol (µg/dL) | 1.56 | 1.2-4.1# |
| T3 (ng/mL) | 1.23 | 0.81-2.58# |
| T4 Thyroxine (µg/dL) | 13.3 | 8.5-24.2# |
| Testosterone (ng/mL) | 0.14 | <1 (immature male)^ |
|  |  |  |
| **Urinalysis** |  | **Reference Data (catheterized samples)†** |
| Color | light yellow | light to medium yellow |
| Clarity | hazy | clear to hazy |
| pH | 6 | 5.6-6.6 |
| Specific gravity (SP) | 1.020 | 1.020-1.033 |
| Glucose (mg/dL) | negative | negative |
| Bilirubin | negative | negative/1+ to 2+ possible |
| Ketones (mg/dL) | negative | negative |
| Blood | moderate (hemolyzed) | negative/up to 2+ from catheterization |
| Protein (mg/dL) | negative | negative/0-2+ |
| Urobilirubin (U/dL) | 0.2 | 0-0.2 |
| Nitrites | negative | negative |
| Leukocytes | negative | negative |
| WBC/HPF | 0 | 0 |
| RBC/HPF | 0 | 0-5 |
| Squamous epithelial cells/HPF | 0 | 0-10 |
| Renal cells/HPF | 0 | 0 |
| Casts | 0 | absent/rare hyaline |
| Crystals | 0 | absent/small amount of calcium oxalate dihydrate |
| Sperm | 0 | absent to TNTC |
| Bacteria | 0 | absent/occasional |
| Sediment | large amount, amorphous | absent/small amount possible |
| Debris | 0 | absent/small amount possible |
| Fat Drops | 0 | 0 |
|  |  |  |
| **Point of Care Blood Analysis** | **T1, T2** | **Reference Intervals (handheld analyzer)◆** |
| pH | 7.29, 7.35 | 6.90-7.46 |
| PCO2 (mmHg) | 63.5, 58 | 47.7-59.1 |
| Glucose (mg/dL) | 95, 145 | 66-141 |
| Lactate (mmol/L) | 4.27, 2.72 | 0.5-1 |
| Note: Reference range sources are indicated in the column header unless otherwise specified next to individual values. TNTC = too numerous to count. T1, T2 = Time point 1, time point 2. CG4+ cartridges on an i-STAT 1 System handheld analyzer (Abbott Point of Care, Princeton, NJ, USA) | | |
| * 1 |  |  |
| ∮ 2 |  |  |
| ^^ 3 (adults) |  |  |
| ** 4 |  |  |
| # 5 |  |  |
| ^ 6 |  |  |
| † 7 |  |  |
| ◆ 8 |  |  |

**References**

1. Schwacke LH, Hall AJ, Townsend FI, Wells RS, Hansen LJ, Hohn AA, Bossart GD, Fair PA, Rowles TK. Hematologic and serum biochemical reference intervals for free-ranging common bottlenose dolphins (*Tursiops truncatus*) and variation in the distributions of clinicopathologic values related to geographic sampling site. *Am J Vet Res.* (2009) 70:973–985. doi: 10.2460/ajvr.70.8.973
2. Lauderdale, LK, Walsh, MT, Mitchell, KA, Granger, DA, Mellen, JD, Miller, LJ. Health reference intervals and values for common bottlenose dolphins (*Tursiops truncatus*), Indo-Pacific bottlenose dolphins (*Tursiops aduncus*), Pacific white-sided dolphins (*Lagenorhynchus obliquidens)*, and beluga whales (*Delphinapterus leucas*). *PLoS One.* (2021) 16:e0250332. doi: 10.1371/journal.pone.0250332
3. Goldstein JD, Reese E, Reif JS, Varela RA, McCulloch SD, Defran RH, Fair PA, Bossart GD. Hematologic, biochemical, and cytologic findings from apparently healthy Atlantic bottlenose dolphins (*Tursiops truncatus*) inhabiting the Indian River Lagoon, Florida, USA. *J Wildl Dis.* (2006) 42:447-54. doi: 10.7589/0090-3558-42.2.447
4. Zaias J, Bossart GD, Cray C. Comparison of Agarose Gel Electrophoresis and Capillary Zone Electrophoresis Methods Using Serum from Bottlenose Dolphins (*Tursiops truncatus*). *Aquat Mamm.* (2021) 47:146-53. doi: 10.1578/AM.47.2.2021.146
5. St. Aubin DJ, Ridgway SH, Wells RS, Rhinehart H. Dolphin thyroid and adrenal hormones: circulating levels in wild and semidomesticated *Tursiops truncatus*, and influence of sex, age and season. *Mar Mammal Sci.* (1996) 12:1–13. doi: 10.1111/j.1748-7692.1996.tb00301.x
6. Robeck TR, O’Brien JK, Atkinson S. “Chapter 11 - Reproduction.,” In: Gulland FMD, Dierauf LA, Whitman KL, editors. *CRC Handbook of Marine Mammal Medicine*. Boca Raton: CRC Press (2018). p. 169–208
7. Deming A, Stacy NI, Balmer BC, Sweeney J, Fauquier DA, Wells RS. Urinalysis in free-ranging bottlenose dolphins (*Tursiops truncatus*) from Sarasota Bay, Florida: (2012) https://www.vin.com/doc/?id=6699186
8. Sharp, SM, Gomez, FM, Meegan, JM, Rowles, TK, Townsend, F, Schwacke, LH, Smith, CR. Using blood gas analysis and capnography to determine oxygenation status in bottlenose dolphins (*Tursiops truncatus*) following the Deepwater Horizon Oil Spill. *Toxics.* (2023) 11:1-18. doi: 10.3390/toxics11050423
